# Developing Mobile BIM/2D Barcode-Based Automated Facility Management System

**DOI:** 10.1155/2014/374735

**Published:** 2014-08-28

**Authors:** Yu-Cheng Lin, Yu-Chih Su, Yen-Pei Chen

**Affiliations:** Department of Civil Engineering, National Taipei University of Technology, No. 1 Chung-Hsiao E. Road, Section 3, Taipei 10608, Taiwan

## Abstract

Facility management (FM) has become an important topic in research on the operation and maintenance phase. Managing the work of FM effectively is extremely difficult owing to the variety of environments. One of the difficulties is the performance of two-dimensional (2D) graphics when depicting facilities. Building information modeling (BIM) uses precise geometry and relevant data to support the facilities depicted in three-dimensional (3D) object-oriented computer-aided design (CAD). This paper proposes a new and practical methodology with application to FM that uses an integrated 2D barcode and the BIM approach. Using 2D barcode and BIM technologies, this study proposes a mobile automated BIM-based facility management (BIMFM) system for FM staff in the operation and maintenance phase. The mobile automated BIMFM system is then applied in a selected case study of a commercial building project in Taiwan to verify the proposed methodology and demonstrate its effectiveness in FM practice. The combined results demonstrate that a BIMFM-like system can be an effective mobile automated FM tool. The advantage of the mobile automated BIMFM system lies not only in improving FM work efficiency for the FM staff but also in facilitating FM updates and transfers in the BIM environment.

## 1. Introduction

Facility management (FM) during the operation and maintenance phase of a facility's lifecycle has become an important topic in research and academic studies. Managing the inspection and maintenance information of equipment and facilities contributes to successful FM. Managing the work of FM effectively during the operation and maintenance phase can be extremely difficult owing to the various types of equipment and facilities. Furthermore, it is inconvenient for FM staff to maintain those facilities by relying on paper-based documents. Unlike the manufacturing industry, information technology is limited in its use and its application in construction [1], with human labor conducting most of the management work, which is inefficient and sometimes error-prone [2].

Building information modeling (BIM) is a computable representation of all of a building's physical and functional characteristics and related lifecycle information and serves as a repository of information for building owners and operators that is used and maintained throughout the lifecycle of a building [3]. BIM is an emerging visual communication tool in the architecture, engineering, and construction (AEC) industry. Recently, various BIM applications have been applied during design and construction phases. However, without a maintenance stage application, BIM cannot fulfill the “lifecycle” mission. Although many projects have been implemented for FM with the use of BIM technology, problems and challenges remain in applied BIM technology that need to be solved and improved in practice.

In FM, the staff usually refers to information such as specifications, checklists, maintenance reports, and maintenance records. As FM staff must record inspection and maintenance results in hard copies, there can consequently be significant gaps in data capture and entry. Such means of communicating information between the facility location and the management office are ineffective and inconvenient. According to the survey findings regarding maintenance work on a commercial building in Taiwan [4], the primary problems regarding data capture and sharing during the FM process are as follows: (1) the efficiency and quality are low, especially through document-based media; (2) it is not easy to refer to the relevant detailed information on facilities; (3) there are data reentry problems; and (4) the use of desktops for operating the BIM models cannot be effectively extended to maintenance management services at the facility location. However, few suitable platforms exist to assist FM staff in using an integrated FM information system from the BIM models and in sharing maintenance information directly at the facility's location.

**Figure 1:**
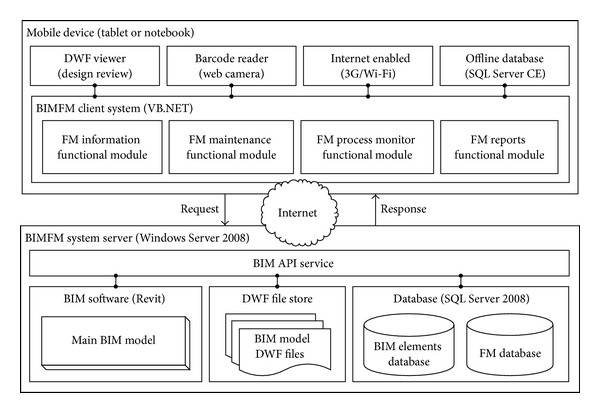
Overview of the BIMFM system framework.

The performance of FM can be enhanced by using Internet technology for information-sharing and communication. In this study, the work of FM includes inspection and maintenance work. By integrating automatic identification technologies (such as two-dimensional (2D) barcode systems), the effectiveness of FM work is enhanced and improved (see Figure 1). In order to enhance the effectiveness of FM work on commercial buildings, this study presents a novel system called the mobile automated BIM-based facility management (BIMFM) system for the acquisition and tracking of maintenance information and provides an information-sharing platform for FM staff that may be accessed with the use of a webcam-enabled notebook or tablet. Integrating BIM and 2D barcode technologies, information, and data entry mechanisms can help to improve the effectiveness and convenience of the information flow in the FM process. The primary objectives of this study include (1) applying BIM and 2D barcode technologies to increase the efficiency of FM data and information collection, (2) directly accessing 2D barcode technologies to link detailed information to the BIM models of facilities, (3) developing a mobile BIM/2D barcode-based system to assist directly the BIM-based maintenance management work at facility locations, and (4) exploring the limitations of the system, addressing problems, and providing suggestions based on the implementation of the case study. The mobile automated BIMFM system is applied to a commercial building in Taiwan to verify our proposed methodology and demonstrate the effectiveness of the FM process in construction. There are two hypotheses in this study: the first is that all BIM models are developed during the construction phase and made ready for FM; the second is that all BIM models must be updated and corrected constantly.

## 2. Related Research Studies

A substantial amount of research has shown the potential of one-dimensional barcode applications in various areas of the construction industry, such as data entry efficiency, labor management, productivity improvement, cost savings, construction equipment and materials tracking, and electronic document management [5–9]. McCullouch and Luepraser [10], for example, illustrated how 2D barcode technology could be applied in the construction industry. Various other research works on the application of barcode models have focused on the integration of other technologies. Navon and Berkovich [11], for example, used barcode and radio frequency identification (RFID) technologies for automated data collection to assist with materials management and control. Shehab and Moselhi [12] illustrated the use of barcode technology to develop an automated system for retrieving engineering deliverables such as drawings, reports, and specifications. Saeed et al. [13] integrated a global positioning system (GPS) with RFID and 2D barcode technologies to provide a solution for pedestrian users that allowed them to access information about buildings and other artifacts.

BIM is changing the traditional construction practices in a broader sense in terms of people, process, working culture, communication, business models, and so forth [14]. Many core benefits, barriers, frameworks, and recommendations for BIM usage are cited in previous work on supporting decisions and improving processes throughout the lifecycle of a project [3, 15–24]. A substantial amount of previous research has examined BIM issues in the operation phase of construction. The Sydney Opera House adopted BIM technology as a means of support for their integrated facility management [25]. Motamedi et al. [26] utilized BIM visualization capabilities to provide FM technicians with visualization that allowed them to utilize their cognitive and perceptual reasoning for problem solving. Becerik-Gerber et al. [27] assessed the status of BIM implementations in FM, the potential applications, and the level of interest in the utilization of BIM by conducting online surveys and face-to-face interviews. Wang et al. [28] not only developed a framework through which one could consider FM in the design stage through BIM but also explored how BIM would beneficially support FM in the design phase. Lin et al. [29] processed different kinds of building components and their corresponding properties to obtain rich semantic information that could enhance applications of path planning in FM. Costin et al. [30] utilized RFID technology for real-time visualization and location tracking in a BIM model. Gheisari et al. [31] explored the ways through which one could integrate BIM with mobile augmented reality (MAR) and make the data accessible through handheld mobile devices in order to enhance current facility management practices.

The BIM approach, which is used to retain facility information in a digital format, facilitates easy updates of FM information in a BIM environment. Although there are many practical applications for using BIM in the maintenance management stage, there are challenges as well. One of the challenges involves the accessibility of the BIM models for FM staff: it usually takes time to refer to and link the corresponding FM element in the BIM model during the maintenance and inspection process [32]. To assist FM staff in obtaining the corresponding BIM model for facilities maintenance management in an automatic and effective manner, this study develops a proposed system that integrates 2D barcode technology to connect automatically to the BIM models. This study then manages facilities by using the 2D barcode technology that is integrated with the BIM approach. By using 2D barcode technology, users can link to the corresponding BIM model of a facility in a quick, automatic, and effective way and access basic information and maintenance problems, while managing FM information during the operation and maintenance phase. Next, the proposed BIMFM system is applied to a case study of a commercial building project in Taiwan to verify its efficacy and demonstrate its FM effectiveness in a BIM-based environment. Finally, the limitations, problems, and suggestions are discussed based on the implementation of the case studies in this study.

## 3. Key Technologies

### 3.1. BIM Technology

BIM is one of the most promising recent developments in the AEC industry [33]. It was developed nearly ten years ago with the aim of providing an environment from which any related information on three-dimensional (3D) entity models could be retrieved during the project lifecycle [14, 34]. BIM is considered essential in AEC for the management, sharing, and exchange of information among project stakeholders such as architects, engineers, contractors, owners, and subcontractors [35], although its technologies are being adopted more slowly in the AEC industry than 2D computer-aided design (CAD) [36, 37]. By enabling visualization of the details of the prospective work, BIM assists construction planners in making crucial decisions [38]. BIM is a new technology in the field of CAD, which contains not only geometric data but also a great amount of engineering data throughout the lifecycle of a building [39]. As a digital tool, BIM supports the continual updating and sharing of project design information [30]. A BIM system enables users to integrate and reuse building information and domain knowledge throughout the lifecycle of a building [40].

There are many BIM commerce tools for creating BIM models (e.g., Autodesk Revit, Trimble Tekla, and the Graphisoft ArchiCAD software). Most of these commerce tools provide software development kits (SDK) for programming purposes. For this study, Autodesk Revit is selected as the main BIM tool because it provides more SDK support than other commerce tools. Furthermore, the use of Autodesk Revit allows the easy export of all of the information regarding a BIM model to a database through open database connectivity (ODBC).

### 3.2. 2D Barcode Technology

Another technology explored as a means of providing accurate and reliable real-time inspection information is the 2D barcode system. The 2D barcode system also has the ability to deliver information on location, including text, audio, and video. Barcode technology was invented in 1950 and it developed rapidly during the subsequent years. With the advantages of higher capacity, lower cost, increased security, traceability, anticorruptibility, and mistake-correcting functionality, the 2D barcode has been widely applied since 1990 [9]. The major characteristics of the 2D barcode are its capacity to represent data content and its arrangement of a specific geometric diagram in a relatively small matrix area that can record significant quantities of data. The 2D Stacked Code and the 2D Matrix Code are the two typical types of barcode classified by their design principle. The 2D Stacked Code was developed based on the one-dimensional barcode. It is composed by thinning down the one-dimensional barcode and stacking it in layers to create multirow symbols. Representative types of the Stacked Code include Code 16 K, Code 49, and Portable Data File 417 (PDF417). The 2D Matrix Code was composed by the distribution of black-white picture elements (square, dot, or other types) in a square area in relative matrix position. Representative types of Matrix Codes include code one, maxi code, quick response (QR) code, and data matrix.

Although many types of 2D barcodes exist, as shown above, the QR code is the most popular type of 2D barcode used in Taiwan. The advantages of the QR code are as follows:

1. high capacity of data content: the QR code can record thousands of characters or numbers, since its capacity is ten times greater than that of the one-dimensional barcode;
2. various data types: the data types stored in the 2D barcode include image, sound, words, and fingerprints, with the capacity for multilanguage expression;
3. ease of production: the scale and shape of the QR code are changeable and are easily made by software and a printer, at a low cost;
4. convenience: the QR code can easily be identified by a mobile phone or a mobile device and is readable in any direction (http://www.qrcode.com/en/about/).

Although RFID technology is suitable for long-distance reading in FM work, the cost of RFID readers and tags is a major problem when many readers and tags are needed. Furthermore, tablets have free software for reading the QR code. Therefore, the QR code is selected and utilized in this study because QR code labels are cheaper than RFID tags.

## 4. System Schematic Design

The 2D barcode has been widely applied in Taiwan. With the advancement of mobile technology, many mobile phones are equipped with cameras, which have the capability to scan 2D barcodes. When a barcode reader program is installed, the user can quickly access product descriptions, web addresses, or e-mail addresses by scanning the barcode. For example, a mobile phone with the Google Android system can read one- or two-dimensional barcodes such as the international article number (EAN), the international standard book number (ISBN), or the QR code after installing the Zxing barcode scanner software. Tablets equipped with cameras also enable the application of 2D barcode scanning to facilitate maintenance management of building facilities. The 2D barcode can be easily identified by mobile devices and record thousands of characters or numbers, since its capacity is ten times greater than the one-dimensional barcode. Furthermore, the 2D barcode's ability to decode mistakes is much higher than that of the one-dimensional barcode [9]. In this study, the main reason for using the 2D barcode is that the brief information and the uniform resource locator (URL) for directly linking the BIM models can be stored within the 2D barcode, unlike the one-dimensional barcode. It is an easy and effective way to link to the BIM models. In this study, we do not adopt an RFID solution because the use of RFID requires RFID tags and an additional RFID reader hardware. The purchasing cost of the RFID tags and the additional RFID reader hardware would be higher than the cost of the 2D barcodes used in this study. Furthermore, 2D barcode labels can be printed without the use of a specialized printer and can be scanned and read by webcam-enabled tablets. Based on the considerations of cost and effectiveness, the 2D barcode is a better choice for implementation. Therefore, this study integrates BIM and 2D barcode technologies to enhance FM work and provide detailed FM information communication. An integrated client/server platform can link all of the information on building facilities to improve the effectiveness of the FM process.

The application of BIM/2D barcode technology in the management of facilities both inside and outside of the buildings focuses on its rapid identification and supports FM staff in handling FM via the 3D BIM models. By scanning the 2D barcode label sticker on a facility, FM staff can obtain the corresponding BIM model of the facility and directly access FM information about the facility, such as instruction manuals, photos, videos of operations, maintenance history, and manufacturer information. Furthermore, a 3D BIM model improves upon the traditional 2D drawings that had difficulty illustrating the vertical location or position of facilities.

The BIMFM system consists of subsystems for BIM, 2D barcodes, mobile devices, and a hub center. The BIM, 2D barcodes, and mobile devices subsystems are located on the client side, while the hub center subsystem is on the server side. Each subsystem is briefly described below.

### 4.1. BIM Subsystem of the BIMFM System

In this study, BIM is used as an information model in the BIMFM system and applied to capture and store information about the facility, including basic descriptions, parameter-related information, maintenance records, and interface reports. Autodesk Revit software was used to create the BIM model files. Autodesk Design Review was used to read the BIM models of facilities. Information integration with the 3D BIM models was achieved using the Autodesk Revit application programming interface (API) and the Microsoft Visual Basic.Net (VB.Net) programming language. The BIMFM system was developed by integrating the 3D BIM models of facilities and maintenance-related information using Revit API programming. ODBC was utilized to integrate the acquired data from different software programs and all maintenance information, such that BIM files, can be exported to an ODBC database for connection with the BIMFM system.

### 4.2. 2D Barcode Subsystem of the BIMFM System

Most people in Taiwan have personal smart phones and tablets and can easily access 2D barcode information. The case study uses the QR code as the 2D barcode system since the QR code reader software is popular in Taiwan and provides the most suitable functionality for facilities maintenance management. The QR code label has a high fault tolerance and its anticorruption capability contributes to longer usage and better identification. One of the major advantages of using the 2D barcode is that no extra cost is required to buy software, since a great number of 2D barcode software applications for tablets are free. Furthermore, all types of 2D barcode labels can be created using a personal computer (PC) printer.

### 4.3. Mobile Devices Subsystem of the BIMFM System

Two mobile devices are used in the BIMFM system. A Samsung Series 7 tablet is used as the webcam-enabled tablet hardware. The Samsung Galaxy Tab runs on Windows 8. All data in the tablet module are transmitted to the server directly through the Internet. An HP Pavilion notebook is used as the webcam-enabled notebook hardware. The HP Pavilion notebook runs on the Windows 7 operating system. All data in the tablet and the notebook are transmitted to the server directly through the Internet via Wi-Fi or third generation (3G).

### 4.4. Hub Center Subsystem of the BIMFM System

The hub center is an information center in the BIMFM system that enables all participants to log on to a hub center and immediately obtain information required for FM. Users can access different information and services via a single front-end access point on the Internet. For example, FM staff can log on to the hub center and securely access the latest FM schedule information. FM managers can check maintenance status, results, and various other inspection-related data. All facilities-related information acquired within the hub center subsystem is recorded in a centralized system database. FM staff can access the required information via the hub center subsystem based on their access privileges.

The amount of maintenance information stored will increase over time if all FM information is recorded in the BIM model. Because BIM models cover a wealth of building information, system storage space should be reserved for crucial information, such as spatial information, facility ID and name of the facility, facility location, and other critical information. In order to keep the system performance at an acceptable level, the information derived by other applications should be stored in an external location. Therefore, two databases are incorporated into the design of the BIMFM system: the BIM elements database and the FM database. The BIM elements database stores only basic information (such as the position, ID, and name of the facility and key parameter information of components). Related maintenance data and information are stored in the FM database.

The accuracy of the BIM model will directly affect FM operations in the BIMFM system. To prevent too many users from simultaneously using the BIM models and, in turn, affecting their accuracy, the BIM engineer can update the information from the BIM elements database directly in the BIMFM system. The latest information in the BIM elements database automatically resyncs when content changes. In this framework, all building facility information from BIM can be saved and updated in the BIM elements database without directly accessing the BIM models.

FM operations do not require all building information; they only require information about necessary maintenance, although the BIM model may cover the whole building. Therefore, during the pre-FM process, the BIM engineer is responsible for determining whether to create the DWF (design web format) file of the BIM model in advance and save it as a source for decomposed BIM models based on the requirements of the FM operations. Not only can the DWF format retain building information, but also its file size is smaller than the general BIM model file. The BIMFM system can be improved with the use of the DWF file for the 3D BIM illustrations, and the system's performance is enhanced for users by reviewing the 3D BIM models. Furthermore, the BIM elements database in the server can store accurate information on the BIM models.

In the BIMFM system, the following three major roles are involved in FM: a BIM engineer, an FM manager, and FM staff. To ensure that the FM operation does not affect the maintenance operation of the BIM model, this study utilizes client-server system architecture. In the BIMFM system, the BIM elements database stores all of the information on the BIM models on the server side. In addition, only BIM engineers are allowed to access and edit the BIM models and export data to the BIM elements database using the BIM software directly on the server side. On the client side, the FM manager and FM staff refer to facility information through the BIM elements database and edit FM information through the FM database in the BIMFM system.

The BIMFM system server supports four distinct layers, each with its own responsibilities: management, data access, application, and presentation. The following section describes these four distinct layers in the BIMFM system.

The management layer provides BIM engineers with the tools to edit and manage BIM models using the BIM software. BIM engineers can access and edit the BIM models saved in the server through the Internet. With the development of the BIM tool APIs, the management layer can not only export data from the BIM models to the BIM elements database but also import data from the BIM elements database to the BIM models. Furthermore, facilities maintenance information can also be recorded in the BIM elements database in the management layer.

The database layer in the BIMFM system consists of two databases: the FM database and the BIM elements database. The FM database stores all facilities maintenance records, while the BIM elements database stores complete facility information, including facility number, name, and type, in the BIM models. The FM database records detailed maintenance information in accordance with the facility ID. The primary key establishes a relationship between the facility ID and the main index. Therefore, information can be used for data association for data mapping to retrieve complete facilities maintenance information based on the facility ID between the two databases.

The application layer defines various applications for the major system and API modules. These applications offer indexing, BIM model data updates and transfers, facility status visualization, and report generation functions. The application layer integrates and uses the BIM software to open the BIM models using developed API modules. Finally, the application layer can automatically acquire data and analyze the BIM models based on a request and then send the results back to the client side.

The presentation layer is the main implementation platform of the BIMFM system. During the FM process, the FM manager and FM staff can use a tablet (client side) and the utilities in the BIMFM system for the FM operation. The presentation layer is integrated with a QR code device, automatically displays the location information of the BIM model, records maintenance information, illustrates the different conditions and status of FM, queries the history, and exports reports on FM results.

## 5. System Development

The BIMFM system server is based on the Microsoft Windows Server 2008 operating system with an SQL Server 2008 R2 as the database. The BIMFM system is developed using VB.NET programming, which is easily incorporated with ADO.NET to transact FM and BIM information with an SQL Server database. The BIMFM system consists of three different user areas, FM staff, FM manager, and BIM engineer areas. Access to the BIMFM system is password-controlled.

### 5.1. System Functionality Description

**Figure 2:**
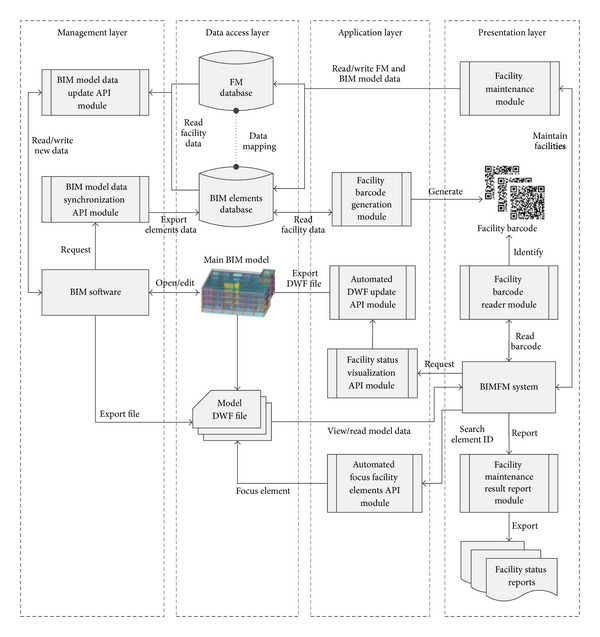
System and module framework of the BIMFM system.

This section describes the implementation of each major functionality module in the BIMFM system (see Figure 2).

#### 5.1.1. FM Information Functional Module

The functional module provides FM staff with detailed FM information on facilities by reviewing 3D BIM models. This module enables all FM staff to refer to related FM information and historical maintenance records for the selected facility quickly and easily in the 3D BIM-based environment. This module allows FM staff to refer to basic information and specifications associated with 3D BIM models during the FM process. This module also has a search function that enables the information to be found and retrieved easily.

#### 5.1.2. FM Maintenance Functional Module

FM staff can download up-to-date maintenance records through the 3D BIM models and enter facility maintenance results directly into the 3D BIM models. Additionally, the module can automatically produce the corresponding maintenance forms through the 3D BIM models. Tablets display the checklist for every facility maintenance task. FM staff can record maintenance information such as dates, conditions, inspection results, descriptions of problems that have arisen during maintenance, and recommendations. Furthermore, FM staff can also check tasks that do not pass the inspection and select relevant tasks from lists in the 3D BIM models. One of the benefits of the module is that maintenance results and records can be transferred between a tablet and the BIMFM system by real-time synchronization, eliminating the need to enter the same data more than once.

#### 5.1.3. FM Process Monitor Functional Module

This functional module is designed to enable FM managers to monitor the FM process. The process monitor module provides an easily accessed and portable environment where FM staff can trace and record all maintenance information and status through the visualized and colorized BIM model.

#### 5.1.4. FM Reports Functional Module

Users can easily access the FM reports functional module to identify needs and analyze FM results information. Authorized records for interfaces can be extracted and summarized for the final FM result-related reports. Furthermore, all FM reports can be extracted using commercially available software such as Microsoft Excel.

### 5.2. System API Modules Description

In order to integrate the system with the BIM models, the following API modules are developed in the BIMFM system.

#### 5.2.1. The BIM Model Data Synchronization API Module

The main function of this module is to automatically synchronize the latest information on the BIM models with the BIM elements database. Although the BIM software provides the open database connectivity (ODBC) database export function, there are still many required data elements for FM that cannot be exported through this function, such as self-defined parameters information in the BIM elements modules. That functionality is provided by the API. All required information in the BIM models is automatically synchronized to the BIM elements database based on required information for FM by the API development. The module will retain the existing data and update the changed data synchronization if the exported information already exists in the BIM elements database.

#### 5.2.2. Facility Barcode Generation and Reader API Module

This module generates a QR code label automatically and links to the related facility BIM module or BIM element module. Because there are typically thousands of facilities for FM, the FM staff usually requires significant time and effort to make QR code labels for FM purposes. To address this problem, the module can generate the QR code label to obtain basic required information (such as device number and purchase date) and linkage to related 3D BIM modules. By scanning the QR code, users can link and check the related BIM modules directly without spending too much time searching for them. Furthermore, a QR code can be generated and accessed quickly to find available BIM models in the room corresponding to the facility location.

#### 5.2.3. Automated Focus on Facility Elements API Module

This module allows users to access the related BIM models by scanning a QR code label attached to the surface of the facility. When the user scans the QR code label, this module will automatically identify the facility or facility location based on the corresponding floor to open the corresponding BIM models file (in DWF format). If the facility is positioned too high up to scan its QR code label, the user may scan the QR code label attached to the entrance of the room and directly select and access the corresponding BIM models in the room.

#### 5.2.4. BIM Model Data Update API Module

This module provides the functionality for updating facility information from the FM database to the main BIM model automatically. The DWF format of BIM models in the BIMFM system allows users to view and access BIM models without changing any information in the BIM models. When FM-related information changes and requires feedback to the BIM model (e.g., the facility is lost or scrapped), the most recent maintenance date and the facility replacement date can be automatically updated for the corresponding BIM model. Therefore, BIM engineers and FM managers can directly access the updated facility maintenance information in BIM software.

#### 5.2.5. Automated Updated DWF File API Module

This module is mainly to allow users to quickly access the latest BIM models in the BIMFM system through the updated whole or separated DWF file. When the size of BIM models increases, system performance slows down. A solution is to decompose whole BIM models into smaller BIM models (exported as separate DWF files) for improved system performance. Models may be decomposed according to a floor or a specific area. Furthermore, the module will update the separated DWF file automatically when any information changes in the BIM model.

#### 5.2.6. Facility Status Visualization API Module

The module provides the visualization functionality for FM status through a visualized BIM model. Through a systematic FM analysis of test results, the module displays different colors to illustrate various conditions and FM status (such as qualified inspection, required repair status, and obsolete facility). Users can access the overall different maintenance conditions and FM status quickly through the visualized BIM model.  Table 1 displays the colors associated with each status.

There are two subsystems in the BIMFM system. The first subsystem is the API monitoring subsystem for BIM engineers located on the server side. This subsystem deals with integration services of BIM models in the BIMFM system. These services include BIM elements database initialization, updating facility maintenance information, and visualizing the maintenance status of facilities. Another subsystem is the maintenance subsystem located on the client side. This maintenance subsystem is developed for FM staff and FM managers to deal with FM operations in the facility's location, such as reading the barcode attached to the facility, recording FM, and reporting FM results.

### 5.3. System Process Description

**Figure 3:**
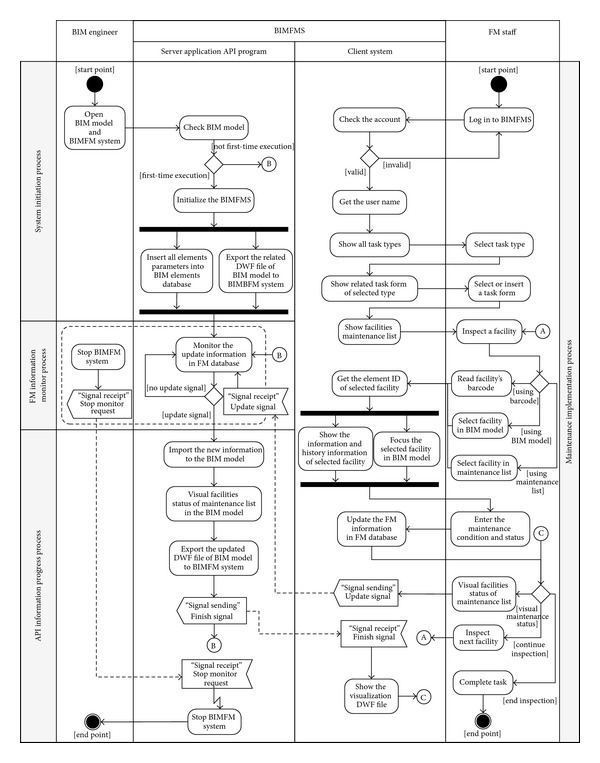
The system process flowchart used in the BIMFM system.

There are four processes used in the BIMFM System including the system initialization process, FM information monitoring process, maintenance implementation process, and API information processing process (see Figure 3).

#### 5.3.1. System Initialization Process

The purpose of the system initialization process is to provide adequate information on a facility for maintenance operations. The BIM model must provide all information and related models (DWF files) on a facility as an information requirement for facility maintenance operations. When the BIM model is input with complete facility information, the BIM engineer needs only to use BIM software (such as Revit) to open the BIM model and run the BIMFM API monitoring system to complete the setup work. When the BIM engineer opens the API monitoring system, the system will automatically determine whether the BIM model is run in the program for the first time. If so, the system will automatically insert all the facility elements information into the BIM elements database and the BIM 3D model (such as the model of each floor and a special area model) for exporting to the DWF file in the BIMFM system. If not, the system will only automatically update any new facility elements information to the BIM elements database and update the changed DWF file.

#### 5.3.2. FM Information Monitoring Process

When the system initialization process is completed, the system will automatically enter the FM information monitoring process. The major purpose of the FM information monitoring process is to check and track whether the user requests the server to update API information. When the demand signal is transmitted to the system by a user, the system will begin to update API information again in the server. Furthermore, the BIM engineer can stop the FM information monitoring process at any time. All API services in the application layer of the BIMFM system will stop operation when the FM information monitoring process is stopped.

#### 5.3.3. Maintenance Implementation Process

During the maintenance implementation process, the maintenance list varies according to the maintenance task categories. The design lets FM staff work on maintenance operations effectively according to the task categories and maintenance list. FM staff can utilize the webcam-enabled Tablet PC to access the BIMFM system and show all the task categories and maintenance lists based on different levels of access. After FM staff selects a particular task category, the system shows the history task form for that category. FM staff can view the other task forms, edit the unfinished task form, or add a new task form. When FM staff selects or adds a task form, the system retrieves facility information from the BIM elements database based on the task types. Furthermore, a list of all related maintenance and results will be illustrated with the BIM model for FM work preparation. FM staff can access inspection information and the maintenance status effectively. During the maintenance implementation process, FM staff can use the system directly and read the QR code attached to the surface of the facility. When the system receives the facility ID, the system automatically displays the facility's basic information and historical maintenance data in the BIM model. Furthermore, the facility's BIM model will be selected, focused, and highlighted using different color. User can obtain basic information on the facility by scanning the QR code attached to the facility, clicking the BIM model, or selecting from a maintenance list. After selecting the facility through one of the three methods, the FM staff can handle maintenance work and record the status and result of maintenance. Finally, all maintenance records and information are stored in the FM database.

#### 5.3.4. API Information Processing Process

During the process of maintenance operations, the maintenance status can be enhanced by color visualization in the BIM model through the API information process. Through the functionality that visually depicts the status of maintenance list items, the system will send the updated signal automatically to the server side of the BIMFM system. The system will start API information processing if the BIMFM system is running during the FM information monitoring process. First, the system will get related maintenance information from the maintenance list in the FM database and update the main BIM Model through API information processing. After the maintenance list is updated in the database, API creates a new 3D view automatically; the 3D view assists with color visualization of the facility based on the maintenance status. The 3D BIM model of facilities in the selected task form will be displayed in a color based on maintenance status, while the rest of the BIM model elements will be displayed in translucent white to enhance the visualization effect. All color visualization is described in Table 1. Finally, the system will export the completed 3D view BIM model to the DWF format automatically, store DWF files in the server side, and return the completed signal to the client side. The system will automatically connect to the server and open the visual DWF files to assist in the visual effect of the maintenance status when the client receives the completion signal.

### 5.4. System Information Flow Description

**Figure 4:**
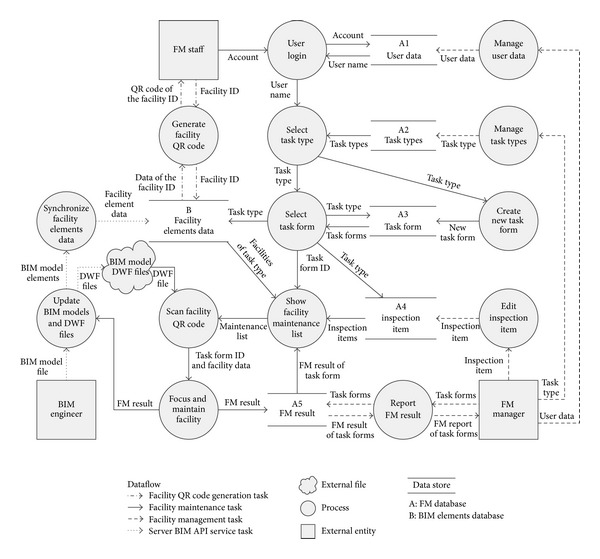
The information flow used in the BIMFM system.

There are three external entities in the BIMFM system. They are entity* BIM engineer*, entity* FM staff*, and entity* FM manager* (see Figure 4). This section describes each external entity in the BIMFM system.

The entity* BIM engineer* is primarily responsible for starting the BIMFM system in the BIM API service on the server side and opening the applied BIM model in the system. This is called the BIM API service task in the study. In the beginning, entity* BIM engineer* must use the BIM software to open the BIM model and also start the BIM API service. When the BIM model is imported to the BIMFM system, the process* update BIM models and DWF files* will automatically update the BIM model information (including BIM model elements and the DWF files). The process* synchronize facility elements data* will check the updated facility element data first. The needed updated information will be imported into the* store facility elements data* in the BIM elements database. All updated DWF files will be saved directly in an external file.

The entity* FM manager* is primarily responsible for handling the BIMFM planning and management work. The operation is called the FM task in the study. The FM tasks include various operations and procedures of management (including processes such as* manage user data*,* manage task types*, and* edit inspection item*). The entity* FM manager* can handle all system data management during the processes. All changed and updated information will be imported into the corresponding data store of the FM database. Furthermore, the entity* FM manager* can select multitask forms to export as a report. The process* report FM result* will acquire the related information based on selected task forms from table* FM result data* of the FM database. Furthermore, the process* report FM result* analyzes the information, compiles it into report format, and sends back a final report to the entity* FM manager*.

The entity* FM staff* is primarily responsible for handling the facility QR code generation task and facility maintenance task. The facility QR code generation task is developed to handle the preparation of QR code work. Entity* FM staff* may create one QR code label or a set of QR code labels for FM use. The process* generate facility QR code* will obtain related information on the facility from the* BIM elements data* table based on the facility ID, send the information for QR code coding, create the QR code image file for the facility, and send it back to entity* FM staff*. The facility maintenance task handles various operations and procedures of FM. In the beginning, the process* user login* will check the user's authority based on the user name and password in the store* user data*. The process* select task type* will show task types in the store* task types* for the selection if certification is passed. The process* select task form* will show the related FM task form based on task type for FM staff selected. If the maintenance work is a new activity, the process* create new task form *will create a new task form based on the selected task type and save it in thetable* task form data*. After entity* FM staff* selects a task form, the process* select task form* will acquire the necessary facilities list from the table* facility elements data* in the BIM element database and acquire necessary inspection items from table* inspection item data* in the FM database. The facilities list and inspection items integrated with the selected task form ID will be imported into the show process* facility maintenance list*. Furthermore, the show process* facility maintenance list* acquires maintenance results from table* FM result data* in the FM database. The process* show facility maintenance list* will arrange information as maintenance list and export information to the process* scan facility QR code* after acquiring complete information. When entity* FM staff* scans the QR code of the facility, the facility QR code process will decode the QR code automatically to send task form ID and facility data into the process* focus and maintain facility*.

The process* focus and maintain facility* will zoom in and highlight the BIM model of the facility automatically when it receives the facility data. Final maintenance results will be updated in the table* FM result data* in the FM database when entity* FM staff* finishes the maintenance work and sends back the process* update BIM models* to update facility status in main BIM model. The process* update DWF files* in the BIM API service will update all changed DWF files automatically, save them in the external file, and send DWF files back to the process* scan facility QR code* to let FM staff review the visualized DWF file if FM staff requests facility status visualization.

**Figure 5:**
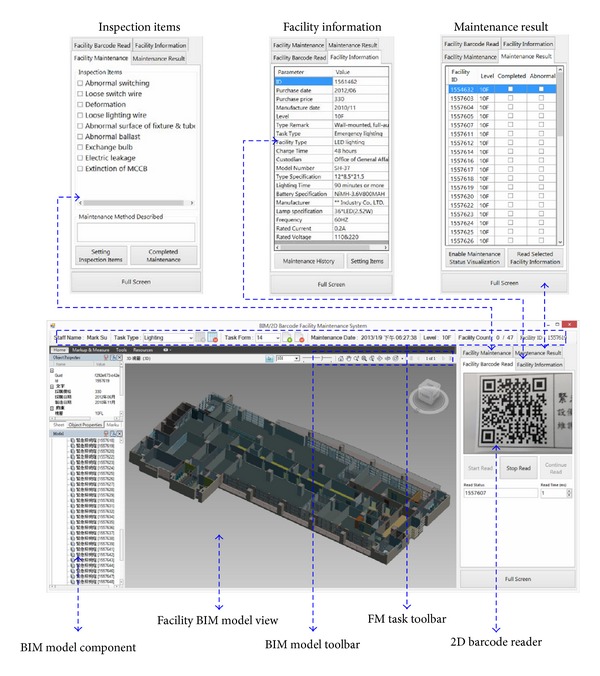
GUI of the BIMFM system.

Integrated with the above design concept, more complex operating procedures of FM are simplified and developed in the BIMFM system. One of the major characteristics of the BIMFM system is to provide users with easy-to-use visualization for handling FM work. By clicking the list, each task form will show the list of facilities requiring maintenance, historical maintenance information, and the status and condition of facilities maintenance. By scanning the QR code attached to the facility, the corresponding BIM models are linked and illustrated quickly and effectively in facility location. Finally, all maintenance results are sent back and saved in the main BIM model. The proposed approach provides a means to update the facility information of the BIM model and FM information synchronization. Finally, in order to let FM staff applies the system easily and effectively, the layout of the system is designed based on FM staff's suggestions.  Figure 5 shows the graphical user interface (GUI) of the BIMFM system.

## 6. System Validation

### 6.1. Case Study

For the case study, the proposed BIMFM system of this study was applied to a building in Taiwan; that is, a BIMFM system was utilized for the FM of the case study building, which contained approximately 20 facilities that had to be managed and inspected. Usually, the FM work was executed every month. Existing approaches for tracking and managing the FM work relied on paper-based records. The bulk of the FM work was paper-based and documented by repeated manual entry, although an FM system was developed for a standalone software application. Therefore, the FM staff in the FM division utilized the BIMFM system to enhance the FM work in the case study.

**Figure 6:**
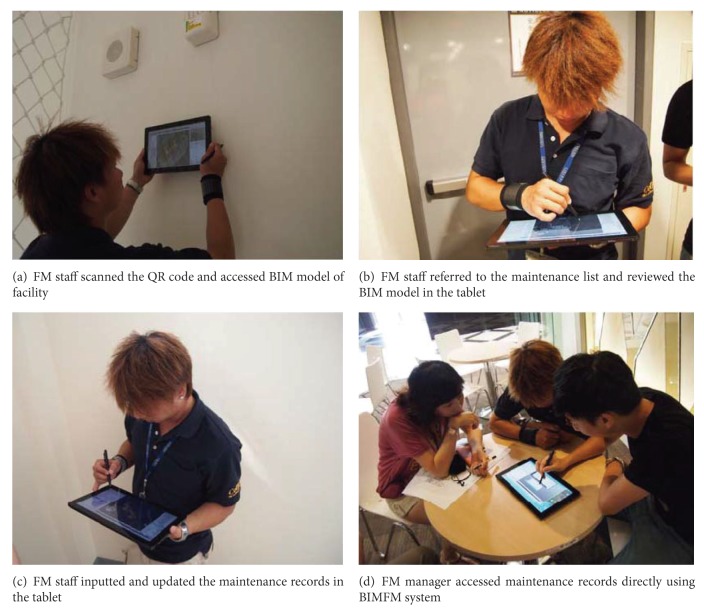
FM staff using webcam-enabled tablet to scan QR code label for FM work in the case study.

After the critical facilities were selected for FM work, each QR code label was made, and the unique ID for each facility was entered into the BIMFM system database for quick search. During the FM process, the QR code label was scanned for basic information about the facility before the FM work started. Before the FM work began, the FM staff could check the facility list from webcam-enabled tablets, refer to the relevant information, and begin preparation work without printing any paper documents. During the FM process, the FM staff scanned the QR code label first (see Figure 6(a)). The BIMFM system showed the basic information and the BIM model of the facility after scanning the QR code label. The FM staff could then check further detailed information like maintenance instructions, notifications, and accessories list, all of which were supported by BIMFM (see Figure 6(b)). After the FM work, the FM staff entered the results of maintenance, edited the description in the tablet, and provided the updated information to the system (see Figure 6(c)). When a facility required repairs, the system also provided the manufacturer's problem information for immediate reference. Finally, the facilities manager and the authorized FM staff accessed the updated information simultaneously from their offices (see Figure 6(d)).

### 6.2. Evaluation and Results

Overall, the field test results indicate that the integration of BIM and 2D barcode labels is an effective tool for the FM of a building. All 2D barcode labels survived use in the pilot test over the two-month testing period. Approximately 25 users participated in field trials of the FM process. The BIMFM system was installed on the main server in the FM division of the building.

During the field trials, verification and validation tests were performed to evaluate the system. The verification test aims to evaluate whether the system operates correctly according to the design and specification, while the validation test assesses the usefulness of the system. The verification test was carried out by checking whether the BIMFM system could perform the tasks specified in the system analysis and design. The validation test was undertaken by asking selected case participants to use the system and provide feedback by answering a questionnaire. Twenty-five participants were involved in the evaluation test. To evaluate the system function and the level of satisfaction with the system's capabilities, the users of the system were asked to grade the conditions of system testing, system function, and system capability, separately, in comparison with the typical paper-based FM approach. Some comments for future improvements to the BIMFM system were also obtained from the case participants through the user satisfaction survey. Finally, Table 2 shows a comparison of the current approach and the proposed system.

The percentage of satisfied users (96%) obtained from the user satisfaction survey indicates that the BIMFM system is quite adaptable to current FM practices in a building and is attractive to users. The overall result implies that the BIMFM system is considered well designed and is able to enhance current time-consuming FM processes. The satisfaction rate exceeding 88% also indicates that the visual BIM model that provides FM support is very helpful. The 92% rate of satisfaction with the integration of the QR code in the BIMFM system for the direct access of the BIM model also indicates that this integration of the QR code is considered effective and necessary. Moreover, no additional work is required to complete the documentation beyond the data collection process. The advantages and disadvantages of the BIMFM system identified from the pilot study are identified.

In the cost analysis, the total cost of the equipment applied in this study was $3,500 US dollars (including an 11-inch webcam-enabled tablet and one PC server). Most personal computers can generate and print QR code labels using free software. Furthermore, there is no additional cost for the QR code reader hardware because most tablets are equipped with cameras that enable 2D barcode scanning. The experimental results demonstrate that the BIMFM system can enhance the visual FM process significantly and effectively when using a BIM approach that is integrated with 2D barcode technology. The use of these technologies significantly improves the overall performance of maintenance operations.

### 6.3. Limitations and Barriers

The findings of this case study revealed several limitations of the BIMFM system. The following are inherent problems recognized during the case study.

- It is difficult for new users to operate the BIM model in the BIMFM system. Some FM staff are initially unfamiliar with BIM models. As it usually takes time to learn how to use BIM models, the use of the BIM system in the case study initially lengthened the FM operation over the traditional approach, since users required time to find the corresponding BIM model and fill out the FM information in the BIMFM system. After the user becomes skilled and familiar with the BIM model, the time required by the current approach and the proposed system becomes almost exactly the same as in the previous FM operations.
- If BIM models do not exist for the purpose of construction management during the construction phase, the BIM approach integrated with FM will not likely be implemented within the BIM environment. Most FM companies do not want to spend the required time and cost to use BIM for only FM work on building projects.
- As QR code labels attached to outdoor facilities are easily damaged because of external environmental pollution (such as dust and rain), it is necessary to consider and enhance the protection and the waterproofing of the QR code labels.
- The QR code technology's short-read distance range was the primary limitation in the case study. In some facilities, the QR code labels were installed up high where FM staff could not easily reach them. Therefore, it is recommended that the QR code label be attached at a lower space on the facility for easier scanning or, alternatively, that the QR code label be associated with the BIM models for all of the facilities in the room and placed at the entrance of the room. A user can then scan the QR code label at the entrance of the room to find and select directly the BIM model of a facility.
- The best read distance of QR code labels is about 3 meters (in a straight line). Based on the case study, the scanning distance varies depending on the tablet camera's resolution. Furthermore, the webcam-enabled tablet cannot identify a QR code label and read the information if the lighting is too low. The QR code labels cannot be recognized if the corner side of the position-detection pattern block position is damaged or polluted.
- In consideration of the limited storage capacity of the tablet and the notebook, it is suggested that the BIM engineer create the DWF files in the database in advance. However, the file size of the DWF will affect the performance of the BIMFM system directly and evidently. The impact includes the time of reading of the DWF files, the time to search for the facilities, and the smoothness of the system operation. When the DWF file is too large (more than 100 MB), it cannot be opened. Therefore, the main BIM model of the whole building should be exported and separated into many DWF files for each floor. When the FM staff execute the FM work, the system opens the DWF file for the floor only after the FM staff scan the QR code for the facility. Furthermore, the FM staff can quickly refer to the separated DWF files of the floor for FM work.
- Based on the case study, BIM engineers are required to update the BIM models continuously during the maintenance and operation phase. When new equipment or facilities are purchased, the BIM engineers must create an FM element for the new equipment in the BIM model for future maintenance use. Furthermore, communications between the FM maintenance staff and the BIM engineers are necessary and important during the process. The FM staff should inform the BIM engineers about any problems regarding the BIM models. After the BIM engineers correct the BIM models, they must also notify and discuss it with the FM staff. The BIM models require constant maintenance and updates. Another important issue is the quality management of the BIM models. Although the study proposes using the BIMFM system as a means of helping FM staff handle visual facilities maintenance and management work, the advanced management procedures and mechanisms for the quality management of the BIM models for FM must be identified and developed in the future. Particularly, the management mechanisms for updating the BIM models should be developed as the next step of BIMFM system development.

## 7. Conclusions

The BIM approach, which is applied to retain facility information in a digital format, facilitates easy updating of FM information in a BIM environment. Although many practical cases of using BIM during the maintenance management stage exist, one problem is that it is not easy for FM staff to find the corresponding FM element in the BIM model for maintenance management during the phase. In order to assist FM staff with obtaining the corresponding FM element in the BIM model for FM in an automatic and effective manner, this study develops a BIMFM system that integrates 2D barcode technology to connect automatically to the BIM models. The mobile automated BIMFM system not only improves FM efficiency but also provides a real-time service platform during the FM process. In the case study, 2D barcode readings increased the accuracy and the speed of the BIM model searches, indirectly enhancing performance and productivity. The FM staff used webcam-enabled tablets to enhance the FM work seamlessly at facility locations, owing to the system's searching speed and ability to support related information collection and access during the FM process. Meanwhile, on the server side, the mobile automated BIMFM system offers a hub center to provide the FM division with real-time monitoring capacity during the FM process. Integrated with the characteristics of 3D BIM model illustration and BIM parametric design, the mobile automated BIMFM system quickly shows the necessary maintenance information by using a facility's BIM model based on the selected task type and clearly presents the position and the height of the selected facility. The main contribution of this study is to help FM staff obtain the corresponding FM element in the BIM model in an automatic and effective manner by integrating BIM with 2D barcode technology. Furthermore, the proposed solution aims to enhance the tracing and recording of FM status through the visualized and colorized BIM model.

In the case study, the application of the mobile automated BIMFM system helped to improve the FM work of a commercial building in Taiwan. Based on the experimental results, this study demonstrated that BIM technology has the significant potential to enhance FM work. The integration of BIM technology with 2D barcode technology helps FM managers and FM staff to effectively track and control the whole FM process. Compared with current approaches, the combined results demonstrate that a BIMFM system can be a useful mobile BIM/2D barcode-based FM platform. Based on the case study findings, the BIM models must be constantly updated and corrected. Another important issue is the quality management of the BIM models. The advanced management procedures and mechanisms for the quality management of the BIM models for FM need to be identified and developed in the future. Such an endeavor will be the next step in BIMFM system development. Finally, the limitations, encountered problems, and suggestions are discussed based on the implementation of the case studies in this study. Despite the challenges indicated above, the promising results shown in this study demonstrate the great potential of the proposed system as a means of aiding the FM of buildings.

## Figures and Tables

**Table 1:** Description of color usage in BIM model.

Color usage	Description
Green	The facility's maintenance work has been completed and the result is satisfactory.

Red	The facility's maintenance problem has been identified, but the result is not satisfactory.

Yellow	The facility's maintenance work has been out of schedule and the facility's maintenance work has not yet started.

Blue	The facility's maintenance work has been out of schedule and the facility's maintenance work has not been completed.

**Table 2:** Comparison of current approach and proposed system.

Item	Current approach		Proposed approach	
	Method	Average time	Method	Average time
Edit the defect problems of the facility	Edit the defect problems by paper-based sheet	12–20 sec	Edit the defect problems through the BIMFM system	6–12 sec

Find basic information on facility for reference	Review maintenance data on paper-based sheet	12–23 sec	Access basic information on a facility directly by accessing and clicking BIM model	7–13 sec

Refer to relevant historical maintenance information	Refer to paper-based maintenance lists and reports	12–18 sec	Click BIM model and refer to historical maintenance information directly	6–12 sec

Maintenance information updating	Identify and record results on a checklist, then re-enter at the office	42–52 sec	Real-time data entry in the system during maintenance process	22–42 sec

Mark the inspection problems at the facility location	Refer the paper-based maintenance condition and status sheet	1–1.5 min	Illustrate overall maintenance conditions and status of FM quickly through visualized BIM model	40–50 sec
